# TLR4 mutation reduces microglial activation, increases Aβ deposits and exacerbates cognitive deficits in a mouse model of Alzheimer's disease

**DOI:** 10.1186/1742-2094-8-92

**Published:** 2011-08-09

**Authors:** Min Song, JingJi Jin, Jeong-Eun Lim, Jinghong Kou, Abhinandan Pattanayak, Jamaal A Rehman, Hong-Duck Kim, Kazuki Tahara, Robert Lalonde, Ken-ichiro Fukuchi

**Affiliations:** 1 Department of Cancer Biology and Pharmacology, University of Illinois College of Medicine at Peoria, P.O. Box 1649, Peoria, IL 61656, USA; 2 Department of Medical Genetics, Third Military Medical University, Chongqing 400038, PR China; 3 Department of Environmental Health Science, New York Medical College, Valhalla, New York 10595, USA; 4 CHUM/St-Luc, Neuroscience Research Center, Department of Medicine, University of Montreal, Montreal, Canada H2X 3J4; 5 Faculté de Médecine et de Pharmacie, Université de Rouen, 76821 Mont Saint Aignan, Cedex, France

## Abstract

**Background:**

Amyloid plaques, a pathological hallmark of Alzheimer's disease (AD), are accompanied by activated microglia. The role of activated microglia in the pathogenesis of AD remains controversial: either clearing Aβ deposits by phagocytosis or releasing proinflammatory cytokines and cytotoxic substances. Microglia can be activated via toll-like receptors (TLRs), a class of pattern-recognition receptors in the innate immune system. We previously demonstrated that an AD mouse model homozygous for a loss-of-function mutation of TLR4 had increases in Aβ deposits and buffer-soluble Aβ in the brain as compared with a TLR4 wild-type AD mouse model at 14-16 months of age. However, it is unknown if TLR4 signaling is involved in initiation of Aβ deposition as well as activation and recruitment of microglia at the early stage of AD. Here, we investigated the role of TLR4 signaling and microglial activation in early stages using 5-month-old AD mouse models when Aβ deposits start.

**Methods:**

Microglial activation and amyloid deposition in the brain were determined by immunohistochemistry in the AD models. Levels of cerebral soluble Aβ were determined by ELISA. mRNA levels of cytokines and chemokines in the brain and Aβ-stimulated monocytes were quantified by real-time PCR. Cognitive functions were assessed by the Morris water maze.

**Results:**

While no difference was found in cerebral Aβ load between AD mouse models at 5 months with and without TLR4 mutation, microglial activation in a TLR4 mutant AD model (TLR4M Tg) was less than that in a TLR4 wild-type AD model (TLR4W Tg). At 9 months, TLR4M Tg mice had increased Aβ deposition and soluble Aβ42 in the brain, which were associated with decrements in cognitive functions and expression levels of IL-1β, CCL3, and CCL4 in the hippocampus compared to TLR4W Tg mice. TLR4 mutation diminished Aβ-induced IL-1β, CCL3, and CCL4 expression in monocytes.

**Conclusion:**

This is the first demonstration of TLR4-dependent activation of microglia at the early stage of β-amyloidosis. Our results indicate that TLR4 is not involved in the initiation of Aβ deposition and that, as Aβ deposits start, microglia are activated via TLR4 signaling to reduce Aβ deposits and preserve cognitive functions from Aβ-mediated neurotoxicity.

## Background

Alzheimer's disease (AD) is a progressive dementing disorder characterized by Aβ-containing amyloid plaques, intracellular neurofibrillary tangles and degenerating neurons in the brain. Most mutations in the Aβ-protein precursor (APP) and presenilin (PS1 and PS2) genes, which are associated with familial AD, increase production of Aβ, particularly the 42-amino-acid form of Aβ (Aβ42) in the brain [1,2]. Aggregated Aβ is thought to be toxic to neurons in the brain and overexpression of APP with these mutations induces AD-like pathology in mice. One of the important consequences of Aβ deposits in the brain is recruitment and activation of microglia. Microglia function as an immunosurveillance cell in the central nervous system and play important roles in maintaining immune homeostasis. Accumulating studies, however, indicate that activated microglia is a double-edged sword. They are able to protect neurons from toxic substances such as aggregated Aβ by taking up and degrading them while activated microglia release proinflammatory cytokines, chemokines, and reactive oxygen and nitrogen species, which can be harmful to synapses and neurons [3-5]. Therefore, it is of great importance to elucidate the mechanism by which these phenotypes of activated microglia are regulated for development of therapeutic strategies.

Toll-like receptors (TLRs) are first-line molecules for initiating innate immune responses. When activated through TLR signaling, microglia/macrophages respond to pathogens and damaged host cells by secreting chemokines and cytokines and express co-stimulatory molecules needed for protective immune responses to pathogens and efficient clearance of damaged tissues [6]. Fibrillar Aβ has been shown to activate microglia via cell surface receptor complexes that involve several toll-like receptors as essential components *in vitro *[7-9]. We previously demonstrated that an AD mouse model homozygous for a nonfunctional (loss-of-function) mutation of TLR4 had increases in diffuse and fibrillar Aβ deposits as well as buffer-soluble and insoluble Aβ in the brain as compared with a TLR4 wild-type AD mouse model (TgAPPswe/PS1dE9 mice) at 14-16 months of age [10]. We also showed that Aβ-induced upregulation of certain cytokines and chemokines in the brain of the same model at 13-15 months of age was mediated by TLR4 signaling [11]. This AD mouse model starts to develop Aβ deposits in the brain at around 5 months of age. However, it is not clear if microglia are activated in the early stages of AD (reviewed in Wyss-Coray [3]). Heneka et al. [12] even suggested that microglia may be activated before any amyloid deposits are formed. Recently, using *in vivo *multiphoton microscopy and 5- to 6-month-old TgAPPswe/PS1dE9 mice, Meyer-Luehmann et al. [13] reported that amyloid plaques formed extraordinarily quickly over 24 hours and that within 1-2 days of appearance of new plaque, microglia were activated and recruited to the site. On the other hand, Yan et al. [14] reported that amyloid plaques appeared and grew over a period of weeks before reaching a mature size in 6-month-old AD model mice. It is unknown if TLR4 signaling is involved in activation and recruitment of microglia and if TLR4 signaling is neuroprotective or harmful at the early stage of AD when Aβ deposits start. Therefore, in this study we investigated Aβ deposition and microglial activation in the TLR4 mutant and wild-type AD mouse models at 5 months of age in order to elucidate a possible role of TLR4 signaling and microglial activation in early stages of AD pathogenesis.

## Methods

### Animals

Pathogen-free transgenic mice of an AD model, TgAPPswe/PS1dE9 mice [B6C3-Tg(APPswe,PSEN1dE9)85Dbo/J, strain name at Jackson] [15], and B6C3F1 mice were purchased from Jackson Laboratory (Bar Harbor, ME). The transgenic mice express chimeric mouse/human APP with the double mutations (K670N and M671L) and human PS1 with a deletion of exon 9 found in familial AD patients. The transgenic mice have been maintained by mating with B6C3F1 mice. C3H/HeJ mice are highly susceptible to Gram-negative infection and resistant to bacterial lipopolysaccharide (LPS) due to a destructive mutation of the TLR4 gene (TLR4^Lps-d^). The TLR4 genotype was determined by polymerase chain reaction (PCR) followed by restriction enzyme digestion with Nla III as described previously [10]. In this study, four experimental groups at the ages of 5 and 9 months were used: 1) homozygous TLR4 mutant TgAPPswe/PS1dE9 transgenic mice (TLR4M Tg), 2) TLR4 wild-type transgenic mice (TLR4W Tg), 3) homozygous TLR4 mutant non-transgenic littermates (TLR4M non-Tg), and 4) TLR4 wild type non-transgenic mice (TLR4W non-Tg) (n = 8-11/group at each age). Half of the mice were deeply anesthetized and perfused transcardially with cold PBS followed by 4% paraformaldehyde and processed for histochemical and immunohistochemical analyses. Half of the mice were euthanized and their brains were processed for biochemical analyses. Nine month-old mice (n = 10 -11/group) were subjected to the Morris water maze test. A separate set of TLR4W (n = 6) and TLR4M (n = 5) Tg mice at 5 months of age were used for extraction of RNA. Another separate set of TLR4W (n = 7) and TLR4M (n = 4) Tg mice at 9 months of age were also used for biochemical analyses (protein and mRNA). All animal protocols used for this study were prospectively reviewed and approved by the Institutional Animal Care and Use Committee of the University of Illinois College of Medicine at Peoria.

### Morris water maze behavioral test

Acquisition of spatial learning in the Morris water maze was assessed during 5 consecutive days. The Morris water maze consisted of a pool (diameter: 112 cm, wall height: 75 cm) filled with water (21°C) at a height of 31 cm. Powdered milk was evenly spread over the water surface in order to camouflage the escape platform (10 cm × 10 cm) made of white plastic and covered with a wire mesh grid to ensure a firm grip. The pool was contained in a room with visual cues such as light fixtures and a ladder. The mice were placed next to and facing the wall successively in north (N), east (E), south (S), and west (W) positions, with the escape platform hidden 1 cm below water level in the middle of the NW quadrant. An overhead video-camera and SmartTM videotracking software (San Diego, CA) were used to estimate path length and escape latencies in 4 trial sessions for 5 days with approximately 20 min intertrial intervals. Whenever the mice failed to reach the escape platform within 1 min, the mice were guided to the platform and remained on it for 5 seconds. The day after the acquisition phase, a probe trial was conducted by removing the platform and placing the mouse next to and facing the N side. The time spent in the previously correct quadrant was measured for a single 1 min trial. After the probe trial, the visible platform subtask was conducted, with the escape platform lifted 1 cm above water level and shifted to the SE quadrant. A 17 cm high pole was inserted on top of the escape platform as a viewing aid. With the exception that the subtest was conducted in a single day, the same procedure was adopted as with the acquisition phase.

### Immunohistochemistry, histochemistry and quantification of Aβ deposits and activated glial cells

Frozen serial sections (5 μm thick) were cut and subjected to immunohistochemistry using the avidin-biotin-peroxidase method (VECTASTAIN ABC Kit). Endogenous peroxidase was eliminated by treatment with 3% H_2_O_2_/10% methanol Tris-buffered saline (TBS) for 20 min at room temperature. After washing with water and 0.1 M TBS (pH 7.4), slides were blocked with 2% bovine serum albumin (BSA) and 2% goat serum in 0.1% triton-X-100 TBS (TBST) buffer for 60 min at room temperature to prevent non specific protein binding. The slides were then incubated with primary antibody 6E10 (1: 2000; Signet Laboratories, Dedham, MA) or CD11b (1:200; Serotec, MCA711, Raleigh, NC) in 2% BSA, 2% goat serum TBST overnight at 4°C. The sections were rinsed in 0.1 M TBST containing 0.1% BSA and incubated with biotinylated secondary antibody anti-mouse IgG (1: 400) for 6E10 or anti-rat IgG (1:200) for CD11b in 2% BSA, 1% goat serum TBST for 1 h at room temperature. Finally, the avidin biotin peroxidase method using 3,3'-diaminobenzidine as a substrate (Vector, Burlingame, CA) was performed according to manufacturer's protocol. For the negative control, slides were processed with isotype control antibodies (mouse IgG1 for 6E10 and rat IgG2b for CD11b) (BD Biosciences, San Jose, CA) to ensure specific staining by primary antibodies. Some sections were counterstained with hematoxylin. Brain sections were also stained with 1% thioflavin S followed by destaining in 70% ethanol for detection of Aβ fibrils.

Histomorphometry for quantification of amyloid deposition and microglial activation was performed using an Olympus BX61 automated microscope, Olympus Fluoview system and the Image Pro Plus v4 image analysis software (Media Cybernetics, Silver Spring, MD) capable of color segmentation and automation via programmable macros. Each brain section was entirely constructed from pictures that were taken using a 10X objective and 1X eyepiece lens in order to ensure no overlap. Six coronal brain sections from each mouse were analyzed, each separated by an approximately 250 μm interval, starting at 1.6 mm posterior to the bregma to caudal. Areas stained by 6E10, CD11b and thioflavin S were measured using Image Pro Plus v4 image analysis software and expressed as a percentage of total hippocampus or neocortex examined.

For double-label fluorescence immunohistochemistry, after quenching autofluorescence by 10 mg/ml sodium borohydride (NaBH4) and/or 0.05% Sudan Black B, brain sections were subjected to double-label fluorescence immunohistochemistry. For 6E10- and CD11b-double-label fluorescence, brain sections were incubated with these antibodies followed by incubation with Alexa Fluoro 594-conjugated chicken anti-mouse IgG antibody and Alexa Fluoro 488-conjugated goat-anti-rat IgG antibody, respectively. For TLR4-, CD11b, CD45-, and GFAP-double-label fluorescence, the sections were incubated overnight with rat anti-TLR4/MD2 antibody (eBioscience, San Diego, CA). After washing, the sections were incubated with chicken anti-rat IgG antibody conjugated with Alexa Fluoro 488 (Invitrogen, Carlsbad, CA) for 2 h. After washing, the same sections were similarly treated with rabbit anti- CD11b (Santa Cruz Biotechnology), anti-CD45 (Santa Cruz Biotechnology) or rabbit anti-GFAP (astrocytic marker: G-9269, Sigma) antibody followed by incubation with chicken anti-rabbit IgG Alexa Fluoro 594 conjugated secondary antibody (Invitrogen). Some sections were stained with CD11b antibody (MCA711) using the avidin-biotin-peroxidase method as described above and then subjected to thioflavin S staining. After washing, the sections were observed under Olympus IX71 automated fluorescence microscope. The pictures were taken through an Olympus DP70 digital camera system.

### Quantification of buffer soluble brain Aβ by ELISA

Using the left cerebral hemispheres, the brain tissues were dounce-homogenized in carbonate buffer (100 mM Na_2_CO_3_, 50 mM NaCl, pH 11.5) containing protease inhibitors [10 μg/ml aprotinin and 1 mM 4-(2-aminoethyl) benzenesulphonyl fluoride hydrochloride (AEBSF)] and centrifuged at 16,000 g for 30 min at 4°C. Protein concentrations in the supernatants were determined by Bio-Rad Protein Assay (Bio-Rad Laboratories, Hercules, CA), and levels of buffer-soluble Aβ were determined by Aβ42 and Aβ40 enzyme-linked immunosorbent assay (ELISA) kits (Invitrogen) according to the manufacturer's protocol. A duplicate sample from each mouse was used for quantification.

### Quantification of cytokine and chemokine mRNA by real-time PCR

The neocortex and hippocampus were separately isolated and soaked in RNA*later*^® ^Tissue Collection: RNA Stabilization Solution (Ambions, Austin, TX) at 4°C overnight and then moved to -80°C. These tissues were homogenized in Trizol reagent (Invitrogen) for isolation of RNA. RNA samples were treated with RNase-Free DNase (Qiagen, Valencia, CA) for 15 min at room temperature, and total RNA was purified using QIAGEN RNeasy columns. Complementary DNA (cDNA) was generated from 2 μg total RNA in a total volume of 20 μl using SuperScript^® ^III First-Strand Synthesis Kit (Invitrogen) according to the manufacturer's protocol. mRNA levels of interleukin (IL)-1α, IL-1β, IL-4, IL-6, tumor necrosis factor (TNF)-α, transforming growth factor (TGF)-β, interferon (IFN)-γ, CCL2 (MCP-1), CCL3 (MIP-1α), CCL4 (MIP-1β) and CCL6 (C10) in the neocortex and hippocampus were determined by real-time PCR using an iCycler Thermal Cycler (Bio-Rad, Hercules, CA). Complementary DNA (cDNA) was amplified using FastStart SYBR Green Master mix (Roche Applied Science, Indianapolis, IN) with primers listed in Table 1. The PCR amplifications were performed as follows: 10 min preincubation at 95°C to activate the FastStart Taq DNA polymerase, 40 cycles of denaturation at 95°C for 15 s, and primer annealing and extension for 1 min at 60°C. PCR product melting curves were examined to confirm the homogeneity of PCR products. mRNA levels of cytokines and chemokines were normalized by subtracting cycle threshold (Ct) values obtained with GAPDH mRNA and expressed as 2 ^-ΔCt ^[ΔCt = Ct (cytokine or chemokine) - Ct (GAPDH)].

**Table 1:** DNA primer sequences for real-time PCR

Gene	Forward primer (5' to 3')	Reverse primer (5' to 3')
IL-1α	AGGAGAGCCGGGTGACAGTA	AACTCAGCCGTCTCTTCTTCAGA

IL-1ß	TGGTGTGTGACGTTCCCATT	CAGCACGAGGCTTTTTTGTTG

IL-4	ACAGGAGAAGGGACGCCAT	GAAGCCCTACAGACGAGCTCA

IL-6	GAGGATACCACTCCCAACAGACC	AAGTGCATCATCGTTGTTCATACA

IL-10	GGTTGCCAAGCCTTATCGGA	ACCTGCTCCACTGCCTTGCT

TNF-α	TCCAGGCGGTGCCTATGT	CGATCACCCCGAAGTTCAGTA

TGF-β	TGACGTCACTGGAGTTGTACGG	GGTTCATGTCATGGATGGTGC

IFN-γ	TGAACGCTACACACTGCATCTTG	GTTATTCAGACTTTCTAGGCTTTCAATG

CCL2	TGAATGTGAAGTTGACCCGT	AAGGCATCACAGTCCGAGTC

CCL3	CCTCTGTCACCTGCTCAACA	GATGAATTGGCGTGGAATCT

CCL4	CCCACTTCCTGCTGTTTCTC	GAGGAGGCCTCTCCTGAAGT

CCL6	GCCACACAGATCCCATGTAA	GCAATGACCTTGTTCCCAGA

### Isolation of CD11b^+ ^splenocytes by flow cytometry

Spleens were individually isolated from 2-month old TLR4M and TLR4W Tg mice. Single cell suspension of splenocytes was prepared by homogenizing a spleen tissue in 10 ml of RPMI 1640 medium and forcing cells through a cell strainer with 70 μm pores. Splenocytes were centrifuged at 200 *g *for 5 min and suspended with 0.8 ml ACK lysing buffer (UAB Comprehensive Cancer Center) to lyse red blood cells. Cell suspension was centrifuged again at 300 *g *for 5 min and final cell pellets were suspended in 1 × PBS containing 1% BSA. Cells were adjusted to 1 × 10^7 ^cells/ml and incubated with 1 μg/ml of PE rat anti-mouse CD11b (BD Pharmingen) at 4°C for 40 min in the dark. Then, cells were washed twice with 1 × PBS containing 1% BSA and centrifuged at 300 *g *for 5 min. The pellets were re-suspended in 1 × PBS containing 1% BSA at a concentration of 1 × 10^7 ^cells/ml. The cells were sorted into CD11b^+ ^and CD11b^-/low ^population by the FACSCalibur System (Becton-Dickinson Bioscience, Rockville, MD).

### Treatment of CD11b^+ ^monocytes with fibrillar Aβ

Synthetic Aβ42 was purchased from Anaspec (Anaspec Inc, San Jose, CA). Fibrillar Aβ was prepared as described previously [16]. The peptide was dissolved in 1 mM hexafluoroisopropanol (Sigma) and then dried under vacuum in a Speed Vac (Savant, Holbrook, NY). The residual peptide was re-suspended in dimethyl sulfoxide to a concentration of 5 mM. Fibrillar Aβ was made by adding 10 mM HCl to a concentration of 100 μM and incubated at 37°C for 24 h. CD11b^+ ^splenocytes from TLRM and TLR4W Tg mice were plated at the density of 2.5 × 10^5 ^cells/ml and incubated with 1 μM fibrillar Aβ for 4 h. Cells were harvested and RNA was extracted in Trizol reagent as described above. Complementary DNA (cDNA) was generated from 1 μg total RNA in a total volume of 20 μl using SuperScript^® ^III First-Strand Synthesis Kit according to the manufacturer's protocol. The experiment was performed in triplicate for each condition. mRNA levels of IL-1α, IL-1β, IL-6, CCL3, CCL4 were determined by real-time PCR as described above.

### Statistical analysis

Data were expressed as mean ± standard error of the mean (SEM). Intergroup differences were assessed by a repeated measures analysis of variance (ANOVA) and two-tailed Student's t-test for normally distributed data. For the probe trial of the Morris water maze, the Mann-Whitney rank sum test was used for comparison. *P *≤ 0.05 was considered statistically significant.

## Results

### TLR4 mutation does not influence Aβ load in the brain of an AD mouse model at 5 months of age but diminishes microglial activation

**Figure 1:**
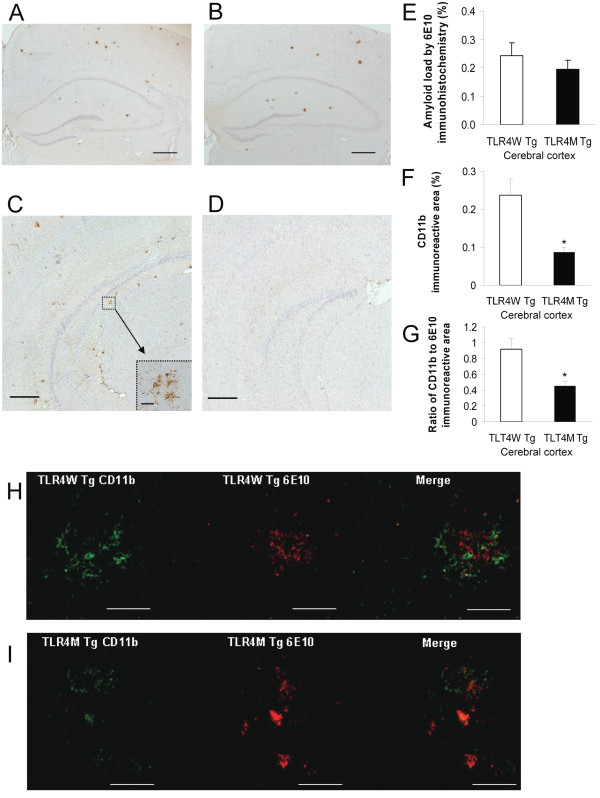
**TLR4 mutation did not influence Aβ load but diminished microglial activation in an AD mouse model at 5 months of age**. Aβ deposits in the brains of TLR4W Tg (A) and TLR4M Tg (B) mice were visualized by anti-Aβ antibody (6E10). Activated microglia are visualized by anti-CD11b antibody in TLR4W Tg (C) and TLR4M Tg (D) mice. The inset image (C) is a higher magnification of the area indicated by the square. (E) Cerebral Aβ plaques are visualized by 6E10 and average percentages of Aβ-immunoreactive areas in the cerebral cortex are shown as a bar graph (means ± SEM). Average percentages of immunoreactive areas in the cerebral cortex for an activated microglial marker, CD11b (F), are shown as a bar graph (means ± SEM). The ratio of CD11b-immunoreactive area to 6E10-immunoreactive area in the cerebral cortex (G) are shown as a bar graph (means ± SEM). Brain sections from 5-month-old TLR4W Tg (H) and TLR4M Tg (I) mice were subjected to double-label fluorescence immunohistochemistry using anti-CD11b (green) antibody and 6E10 (red). *P ≤ 0.05. Scale bars 200 μm for A through D, 50 μm for H and I, and 10 μm for the inset.

TLR4W Tg mice start to develop Aβ deposits in the brain around the age of 5 months. To investigate if TLR4 signaling is involved in initiation of Aβ deposition and microglial activation at the early stage of AD, we determined Aβ load and microglial activation in the TLR4W (n = 5) and TLR4M (n = 7) Tg mice at 5 months. Diffuse and fibrillar Aβ deposits were detected by immunohistochemistry using 6E10 antibody that specifically reacts with human Aβ (Figure 1A and 1B) and were expressed by average percentages of areas showing Aβ immunoreactivity in the cerebral cortex (Figure 1E). No difference was found in Aβ load between TLR4W (0.243 ± 0.045%) and TLR4M (0.196 ± 0.030%) Tg mice (*P *> 0.05).

Activated microglia/myeloid cells in the cerebral cortex were immunostained for expression of CD11b (Mac-1) (Figure 1C and 1D) and the immunoreactive areas were quantified (Figure 1F). CD11b-immunoreactivity (0.237 ± 0.043%) in TLR4W Tg mice was greater than that (0.087 ± 0.011%) in TLR4M Tg mice (*P *= 0.001). The ratio of CD11b-immunoreactive area to 6E10-immunoreactive area in TLR4W Tg mice (0.921 ± 0.133) was greater than that in TLR4M Tg mice (0.459 ± 0.049, *P *= 0.006) (Figure 1G).

To confirm this reduction in CD11b-positive microglia in TLR4M Tg mice, we carried out double immunofluorescence staining of brain sections from Tg mice using anti-CD11b antibody (green) and anti-Aβ antibody (red) (Figure 1H and 1I). Most Aβ deposits in TLR4W Tg mice were closely associated with microglia showing high expression of CD11b and some degree of overlap was found in double immunofluorescence images (Figure 1H), suggesting uptake of Aβ by activated microglia. On the contrary, expression of CD11b in microglia closely associated with Aβ deposits was limited in TLR4 M Tg mice (Figure 1I).

To investigate if the TLR4 mutation alters production of Aβ in the brain, we determined levels of buffer-soluble Aβ40 and Aβ42 by ELISA in early stages of amyloidogenesis. There was no difference between TLR4W (n = 4) and TLR4M (n = 6) Tg mice at 5 months of age in the cerebral buffer-soluble Aβ40 (24.1 ± 3.4 and 27.3 ± 8.6 pg/mg protein, respectively) and Aβ42 (19.1 ± 4.0 and 17.6 ± 7.2 pg/mg protein, respectively) content.

### TLR4 mutation increases Aβ deposits as well as soluble Aβ42 in the brain of an AD mouse model at 9 months of age

**Figure 2:**
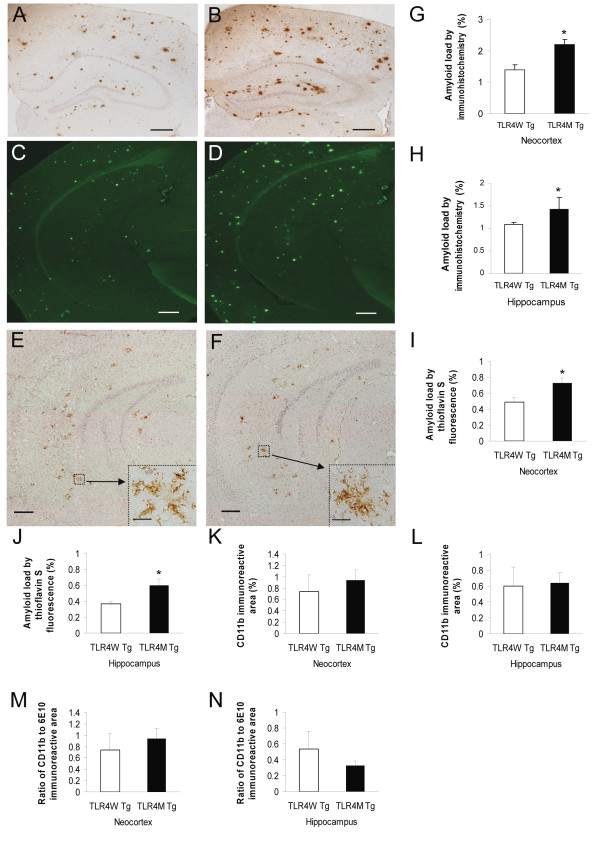
**TLR4 mutation increased Aβ load in the brain of an AD mouse model at 9 months of age**. Aβ plaques in the brains of TLR4W Tg (A and C) and TLR4M Tg (B and D) mice were visualized by 6E10 immunohistochemistry and thioflavin S fluorescence, respectively. Average percentages of areas showing Aβ immunoreactivity and fluorescence in the neocortex (G and I) and hippocampus (H and J) are shown as a bar graph (means ± SEM). Activated microglia were detected by anti-CD11b antibody in TLR4W Tg (E) and TLR4M Tg (F) mice. The inset images (E and F) are a higher magnification of the areas indicated by the squares. Average percentages of CD11b-immunoreactive areas in the neocortex (K) and hippocampus (L) are shown as a bar graph (means ± SEM). The ratio of CD11b-immunoreactive area to 6E10-immunoreactive area in the neocortex (M) and hippocampus (N) are shown as a bar graph (means ± SEM). *P < 0.05. Scale bars 200 μm for A through F and 10 μm for the insets.

We previously reported that cerebral Aβ load increased in TLR4M Tg mice as compared to TLR4W Tg mice at 14-16 months of age [10] but we did not find such an increase at 5 months of age. Therefore, we further examined Aβ load in these AD mouse models at 9 months of age. Diffuse and fibrillar Aβ deposits were detected by immunohistochemistry using 6E10 antibody (Figure 2A and 2B) and Aβ loads were expressed by average percentage of areas showing Aβ immunoreactivity in the hippocampus and neocortex (Figure 2G and 2H). The Aβ loads in the neocortex (2.20 ± 0.15%) and hippocampus (1.42 ± 0.27%) increased in TLR4M Tg mice as compared to TLR4W Tg mice (1.40 ± 0.15%, *P *< 0.005 for the neocortex and 1.08 ± 0.06%, *P *< 0.01 for the hippocampus, n = 6 for each group). Fibrillar Aβ deposits were visualized by thioflavin S fluorescence (Figure 2C and 2D). The Aβ load in TLR4M Tg mice (0.728 ± 0.064% for the neocortex and 0.601 ± 0.080% for the hippocampus) was greater than that in TLR4W Tg mice (0.487 ± 0.056%, *P *= 0.021 for the neocortex and 0.368 ± 0.033%; *P *= 0.026 for the hippocampus, n = 6 for each group) (Figure 2I and 2J).

Activated microglia/myeloid cells in the neocortex and hippocampus were immunostained for expression of CD11b (Figure 2E and 2F) and immunoreactive areas were quantified (Figure 2K and 2L). There was no difference in expression levels of CD11b between TLR4W and TLR4M Tg mice. No difference was found between the two groups in the ratios of CD11b-immunoreactive area to 6E10-immunoreactive area (Figure 2M and 2N).

**Figure 3:**
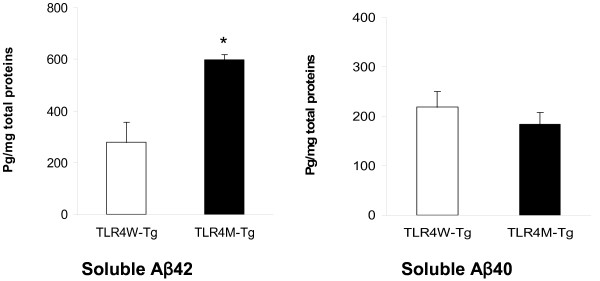
**TLR4 mutation increased cerebral buffer-soluble Aβ42 in an AD mouse model at 9 months of age**. The cerebral buffer-soluble Aβ42 and Aβ40 contents in TLR4W and TLR4M Tg mice were quantified by ELISA. The results are shown as bar graphs (means ± SEM pg/mg total protein). *P < 0.01.

Levels of buffer-soluble Aβ in the cerebrum of TLR4M Tg mice increased at 14-16 months of age as compared to TLR4W Tg mice [10] but not at 5 months of age. Therefore, we further determined levels of buffer-soluble Aβ in the two Tg mouse groups at 9 months by Aβ40- and Aβ42-specific sandwich ELISA. The cerebral buffer-soluble Aβ42 content in TLR4M Tg mice (597.8 ± 21.3 pg/mg protein, n = 4) was significantly higher than that in TLR4W Tg mice (278.3 ± 79.3 pg/mg protein, n = 4, *P *< 0.01) while there was no difference between TLR4M and TLR4W Tg mice in the cerebral buffer-soluble Aβ40 content (183.2 ± 24.5 pg/mg protein and 218.6 ± 32.0 pg/mg protein, respectively, *P *> 0.05) (Figure 3).

### Amyloid plaques are associated with activated microglia expressing TLR4

**Figure 4:**
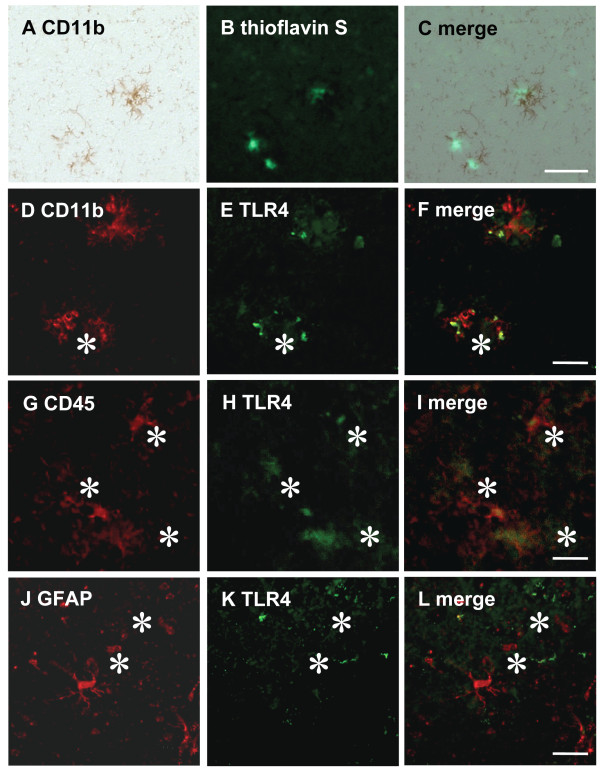
**Aβ plaques were surrounded by activated microglia expressing TLR4**. A brain section from a TLR4W Tg mouse was stained with thioflavin S for fibrillar amyloid deposits (B) and immunostained with anti-CD11b antibody using avidin-biotin immuno-peroxidase method and DAB (A). (C) is a superimposition of (A) and (B), demonstrating that fibrillar Aβ deposits are closely accompanied by CD11b-positive microglia. Brain sections from a TLR4W Tg mouse were labeled with anti-TLR4 antibody (green) (E and H) followed by staining with anti-CD11b (red) (D) or anti-CD45 (red) (G) antibody. The yellow colors in the superimposed images of (F) and (I) between (D) and (E) and between (G) and (H) indicate co-localization of TLR4 with activated microglia (CD11b and CD45), respectively. Overlap between astrocytes detected by anti-GFAP antibody (red) (J) and TLR4 (green) (K) is occasionally found in the merged picture (L). Scale bars 50 μm.Asterisks indicate amyloid plaques.

Aβ deposits have been shown to be closely associated with activated microglia and reactive astrocytes. To assess whether Aβ deposition in the brain can recruit microglia and astrocytes expressing TLR4, we performed a colocalization analysis using anti-CD11b, anti-CD45 (a transmembrane protein tyrosine phosphatase specific for migratory leukocytes including activated microglia), anti-GFAP (an astrocyte-specific intermediate filament protein) and anti-TLR4 antibodies. Brain sections from 9-month-old TLR4W Tg mice were double-stained with anti-TLR4 antibody and 1of 3 antibodis (anti-CD11b, anti-CD45, or anti-GFAP antibody). One brain section was statined with both anti-CD11b antibody and thioflavin S. Almost all fibrillar Aβ deposits stained with thioflavin S were closely accompanied by CD11b-immunoreactive microglia (Figure 4A, B and 4C). Immunofluorescence staining of the brain sections revealed that CD11b- and CD45-positive microglia co-expressed TLR4 (Figure 4D, E and 4F for CD11b, and G, H and I for CD45) whereas limited expression of TLR4 was found in GFAP-positive astrocytes (Figure 4J, K and 4L).

### TLR4 mutation makes an AD mouse model vulnerable to cognitive deficits in the Morris water maze

**Figure 5:**
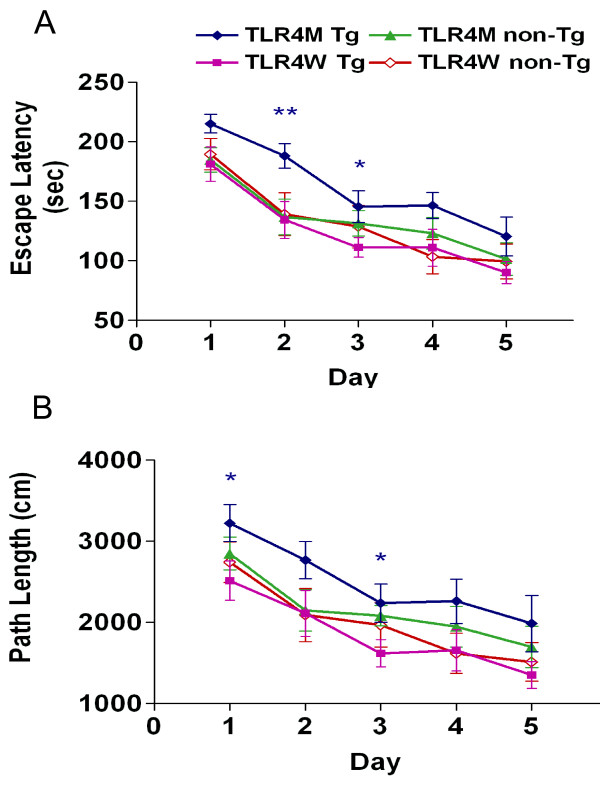
**An AD mouse model with TLR4 mutation was more vulnerable to cognitive impairment as assessed in the Morris water maze**. Total cumulated scores per day for escape latencies (sec) (A) and path length (cm) (B) (means ± SEM) are shown during acquisition of the hidden platform task at 9 months of age. Spatial learning was impaired in TLR4M Tg mice as shown by increases in escape latencies and path length (*P < 0.05; **P < 0.01). No difference was found between any other groups.

We previously reported that the APPswe/PS1dE9 transgenic mice had spatial learning and memory deficits by the Morris water maze at 12 months of age but not at 7 months [17,18]. Because soluble Aβ42 is thought to be neurotoxic and levels of soluble Aβ42 in the brains of 9-month-old TLR4M Tg mice increased compared to those in TLR4W Tg mice, we evaluated the effects of the TLR4 mutation on spatial learning and memory by the Morris water maze in the AD mouse model at 9 months of age. In the acquisition phase, three-way ANOVA with transgene and TLR4 as main factors and days as the repeated measure revealed significant TLR4 (F _(1, 36) _= 5.46, *P *< 0.05) and day (F _(1, 144) _= 19.72, *P *< 0.001) effects for path lengths as well as TLR4 (F _(1, 36) _= 5.50, *P *< 0.05) and day (F _(1, 144) _= 32.84, *P *< 0.001) effects for escape latencies. Paired comparisons by one-way ANOVA revealed higher latencies on days 2 (F _(1, 18) _= 8.33, *P *< 0.01) and 3 (F _(1, 18) _= 4.85, *P *< 0.05) and higher path lengths on days 1 (F _(1, 18) _= 4.58, *P *< 0.05) and 3 (F _(1, 18) _= 4.58, *P *< 0.05) by TLR4M Tg mice (n = 10) than TLR4W Tg mice (n = 10), indicating that mutated TLR4 led to poorer scores than wild-type TLR4 in APP/PS1 transgenic mice. Moreover, TLR4M Tg mice had higher escape latencies than TLR4M non-Tg mice (n = 10) on days 1 (F _(1, 18) _= 5.97, *P *< 0.05) and 3 (F _(1, 18) _= 7.85, *P *< 0.02) (Figure 5A and 5B). No other group differences were found in the acquisition phase or in either the probe or visible platform subtasks. These data indicate that mutated TLR4 impaired acquisition in the early not the late stage of training and spared long-term memory, visual acuity, and swimming abilities.

### Aβ fibrils upregulate expression of certain cytokines and chemokines via TLR4 signaling in the hippocampus and splenic monocytes

**Figure 6:**
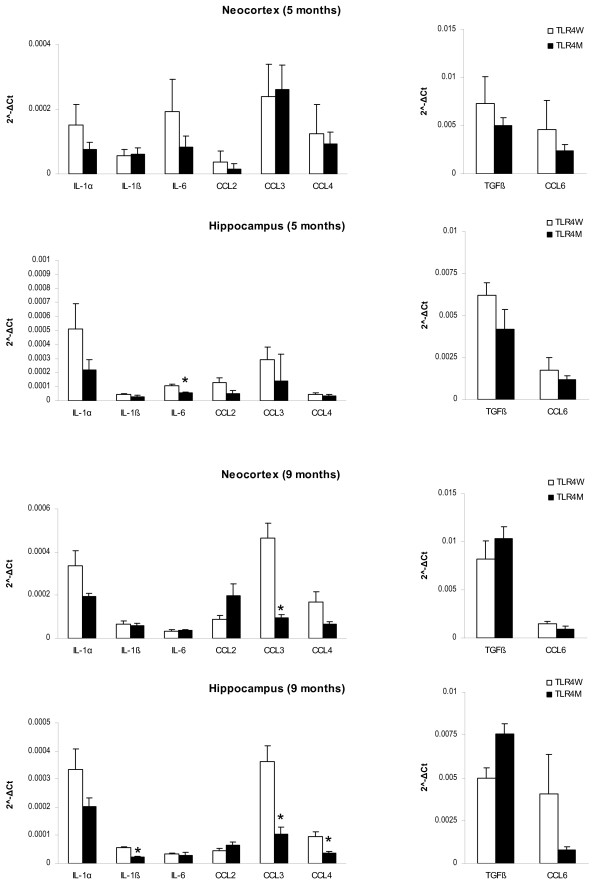
**TLR4 mutation decreased expression levels of IL-1β, CCL3 and CCL4 in an AD mouse model at 9 months of age**. mRNA levels of cytokines and chemokines in the neocortex and hippocampus from TLR4W and TLR4M Tg mice at age 5 and 9 months were determined by real-time PCR using cDNA prepared by reverse-transcription of mRNA. mRNA levels of cytokines and chemokines were normalized by subtracting cycle threshold (Ct) values obtained with GAPDH mRNA and are shown as 2 ^-ΔCt ^[ΔCt = Ct (cytokine or chemokine) - Ct (GAPDH)] (means ± SEM). *P < 0.05.

Induction of TLR signaling by its ligands in macrophages/microglia culminates in activation of transcription factors that upregulate expression of certain cytokines and chemokines, which are required for protective immune responses to pathogens and efficient clearance of damaged tissues. Fibrillar Aβ can activate microglia through interaction with cell surface receptor complexes whose essential components include TLR4 [7-9]. Therefore, we determined levels of cytokines and chemokines in 5- and 9-month-old TLR4M (n = 5 and 4, respectively) and TLR4W (n = 6 and 7, respectively) Tg mice, which are possibly modulated by TLR signaling in the AD pathogenesis. Cycle threshold (Ct) values of mRNAs for the cytokines and chemokines are provided in Table 2. At 5 months of age, mRNA levels of IL-6 in TLR4M Tg mice decreased compared with TLR4W Tg mice in the hippocampus (2-fold, *P *= 0.01) but the difference in the neocortex was not significant (*P *= 0.18) (Figure 6). There were no differences in mRNA levels of IL-1α, IL-1β, TGF-β, CCL2, CCL3, CCL4, and CCL6 between the two groups at 5 months of age. At 9 months, mRNA levels of IL-1β (2.5-fold, *P *< 0.0005), CCL3 (3.5-fold, *P *< 0.05), and CCL4 (3-fold, *P *< 0.05) decreased in TLR4M Tg mice as compared to those in TLR4W Tg mice in the hippocampus (Figure 6). In the neocortex, however, such a decrease in 9-month-old TLR4M Tg mice was found only in the CCL3 mRNA levels (5-fold, *P *= 0.01). Cerebral mRNA levels of IL-4, TNF-α, and IFN-γ were too low to satisfactorily make comparisons under the current experimental conditions.

**Table 2:** Cycle threshold (Ct) values of mRNAs

		IL-1α	IL-1ß	IL-6	TGFß	CCL2 (MCP-1)	CCL3 (MIP-1α)	CCL4 (MIP-1ß)	CCL6 (C10)	GAPDH
**9M** Neocortex	TLR4M	25.7 ± 0.35	28.8 ± 0.49	29.0 ± 0.44	22.3 ± 0.39	27.4 ± 0.74	28.1 ± 0.42	28.6 ± 0.37	23.5 ± 0.50	13.2 ± 0.22
	
	TLR4W	24.5 ± 0.38	27.5 ± 0.30	28.6 ± 0.15	21.7 ± 0.30	27.2 ± 0.33	24.7 ± 0.36	26.2 ± 0.29	22.4 ± 0.23	13.4 ± 0.30

Hippocampus	TLR4M	25.5 ± 0.49	29.8 ± 0.57	29.2 ± 0.41	21.4 ± 0.20	28.03 ± 0.25	27.6 ± 0.34	29.2 ± 0.40	23.1 ± 0.22	12.8 ± 0.33
	
	TLR4W	24.1 ± 0.29	27.4 ± 0.09	28.2 ± 0.13	20.6 ± 0.07	27.7 ± 0.29	24.7 ± 0.37	26.6 ± 0.30	20.7 ± 0.24	12.6 ± 0.35

**5M** Neocortex	TLR4M	29.2 ± 0.50	29.5 ± 0.54	29.1 ± 0.45	23.2 ± 0.41	31.6 ± 0.93	27.4 ± 0.51	28.9 ± 0.42	24.24 ± 0.23	15.5 ± 0.45
	
	TLR4W	27.5 ± 0.20	28.9 ± 0.24	27.1 ± 0.28	21.9 ± 0.10	29.5 ± 0.83	26.8 ± 0.13	27.7 ± 0.44	22.6 ± 0.29	14.6 ± 0.43

Hippocampus	TLR4M	26.2 ± 0.34	29.2 ± 0.33	28.2 ± 0.06	22.0 ± 0.40	28.3 ± 0.49	26.9 ± 0.62	29.0 ± 0.48	23.8 ± 0.34	14.1 ± 0.22
	
	TLR4W	24.9 ± 0.24	28.4 ± 0.24	27.2 ± 0.15	21.3 ± 0.22	26.9 ± 0.32	25.7 ± 0.54	28.5 ± 0.40	23.2 ± 0.69	14.0 ± 0.06

**Figure 7:**
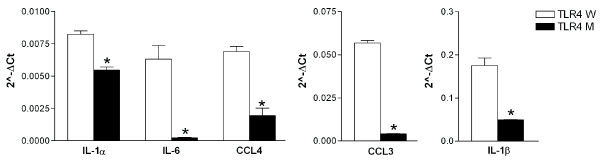
**TLR4M monocytes express less IL-1α, IL-1β, IL-6, CCL3 and CCL4 than TLR4W monocytes after fibrillar Aβ stimulation**. CD11b^+ ^splenocytes (monocytes) from 2-month-old TLR4W and TLR4 M Tg mice were collected by FACS. After treatment with fibrillar Aβ for 4 hours, mRNA levels of cytokines and chemokines were determined by real-time PCR. mRNA levels of cytokines and chemokines were normalized by subtracting cycle threshold (Ct) values obtained with GAPDH mRNA and are shown as 2 ^-ΔCt ^[ΔCt = Ct (cytokine or chemokine) - Ct (GAPDH)] (means ± SEM). *P < 0.005.

Microglia are cells of myeloid origin and considered to be macrophages/monocytes in the central nervous system. Therefore, we have isolated CD11b^+ ^splenocytes (monocytes) from 2-month-old TLR4W and TLR4 M Tg mice by fluorescence-activated cell sorting (FACS). After treating CD11b^+ ^cells with fibrillar Aβ, expression levels of cytokines and chemokines were determined by real-time PCR as described above. Ct values of mRNA for cytokines and chemokines are shown in Table 3. mRNA levels of IL-1α, IL-1β, IL-6, CCL3 and CCL4 in fibrillar Aβ-treated TLR4M CD11b^+ ^cells were significantly lower than those in fibrillar Aβ-treated TLR4W CD11b^+ ^cells (*P *< 0.005 for all measures) (Figure 7).

**Table 3:** Cycle threshold (Ct) values of mRNAs from CD11b positive cells

	IL-1α	IL-6	CCL4	CCL3	IL-1β	GAPDH
TLR4M	27.7 ± 0.10	31.2 ± 0.40	29.3 ± 0.76	28.1 ± 0.13	24.6 ± 0.06	20.2 ± 0.09

TLR4W	25.9 ± 0.08	26.4 ± 0.46	26.2 ± 0.15	23.2 ± 0.06	21.6 ± 0.25	19.1 ± 0.06

## Discussion

Microglial activation and differentiation is complex and can produce diverse phenotypes depending upon their environments, pathogenic contexts, activating ligands and genetic backgrounds [19]. In AD, activated microglia can be beneficial by clearing toxic Aβ assemblies and secreting neurotrophic factors [4,5]. On the other hand, activated microglia can be synapto- and neuro-toxic by initiating and advancing the disease [3-5]. Little, however, is known about the mechanisms by which microglial activation states are orchestrated in AD. Here, we show that a nonfunctional mutation in the TLR4 gene diminished Aβ-induced microglial activation in AD model mice at 5 months of age when the AD model mice start to develop Aβ deposits in the brain. There was no difference in the cerebral Aβ deposits and buffer-soluble Aβ amounts between TLR4W and TLR4M Tg mice in the very early stages of β-amyloidosis. Thus, TLR4 signaling did not alter Aβ production and the onset of Aβ deposition. We also demonstrate that 9-month-old TLR4M Tg mice had increases in the amounts of cerebral Aβ deposits and soluble Aβ42, which were associated with special learning deficits and reduced expression of CCL3. Thus, activation of microglia through TLR4 appears to be neuroprotective.

We previously reported that APPswe/PS1dE9 transgenic mice had spatial learning and memory deficits at 12 months of age but not at 7 months as assessed by the Morris water maze [17,18]. In line with these observations spatial learning deficits were not found in TLR4W Tg mice at 9 months of age but were apparent in TLR4M Tg mice of the same age. Thus, mice with the TLR4 mutation appeared more vulnerable to cognitive deficits associated with the APPswe/PS1dE9 transgenes. The cognitive deficits in 9-month-old TLR4M Tg mice may be attributable to an increase in the cerebral Aβ load, particularly soluble Aβ42. Aβ42 is thought to be more pathogenic than Aβ40 and an increase in the Aβ42 to Aβ40 ratio stabilizes toxic soluble Aβ oligomers [20]. Soluble oligomeric Aβ species have been identified as synapto- and neurotoxic forms of Aβ rather than insoluble amyloid fibrils [21-23]. Soluble oligomeric Aβ levels are elevated in the brains of AD patients and correlate with cognitive dysfunction [24]. Meyer-Luehmann et al. [13] hypothesize that amyloid plaques act as a local source of soluble Aβ causing neuritic alterations. It is thus tempting to hypothesize that activated microglia through TLR4 ligation protect neurons from toxic oligomeric Aβ which is released or produced from amyloid plaques by clearing Aβ oligomers and deposits.

We found an increase in the cerebral Aβ load in TLR4M Tg mice at 9 months of age but not at 5 months. Microglial activation associated with Aβ deposits diminished in TLR4M Tg mice at 5 months. Our results are concordant with the observations from *in vitro *experiments by several other groups [7,9,25] that fibrillar Aβ activates microglia through interaction with its cell surface receptor complex to facilitate Aβ phagocytosis and further, that TLR4 is required for fibrillar Aβ-induced activation of microglia as part of the receptor complex *in vitro*. However, there is no difference in the Aβ load between TLR4W and TLR4M Tg mice at 5 months when amyloid deposition starts. Aβ clearance by activated microglia *in vivo *may be slow as suggested by Meyer-Luehmann et al. [13]. Thus, the difference in the Aβ load may be indiscernible in early stages of Aβ deposition and gradually become evident by 9 months of age.

Fibrillar Aβ activates microglia/monocytes through TLR4 and induces expression of cytokines *in vitro *[7,9,24]. In spite of diminished microglial activation detected by CD11b expression in 5-month-old TLR4M Tg mice, levels of cytokine and chemokine expression were not altered except IL-6 in the hippocampus. Because Aβ deposition starts to develop in this AD mouse model at 5 months, the differences in the cytokine and chemokine levels may be too small to be detected. Alternatively, CD11b-positive microglia do not substantially produce the investigated cytokines and chemokines at early stages of β-amyloidosis *in vivo*.

Expression levels of IL-1β and CCL4 in 9-month-old TLR4M Tg mice reduced in the hippocampus but not in the neocortex. A consistent decrease in both hippocampus and neocortex of 9-month-old TLR4M Tg mice was found only in expression levels of CCL3. CCL3 is a member of the CC chemokine subfamily and its main function is the recruitment of leukocytes to the site of inflammation. Aβ has been shown to induce microglial CCL3 expression and monocyte migration *in vitro *[26-28]. Intrahippocampal injection of Aβ also induces microglial CCL3 expression and transendothelial migration of T cells in rodents [29-31]. Thus, a decrease in CCL3 expression in TLR4M Tg mice may diminish recruitment of bone-marrow derived microglia/monocytes. Bone-marrow derived microglia/macrophages have been shown to be very efficient in restricting the growth of amyloid plaques but resident microglia are not [32]. Furthermore, exercise decreases the cerebral Aβ load in an AD mouse model, which is accompanied by an increase in cerebral CCL3 levels [33]. Here, we demonstrated TLR4 mutation diminished fibrillar Aβ-induced CCL3 expression in monocytes, suggesting that TLR4 signaling may play an important role in recruitment of microglia/monocytes. Therefore, the decrease in CCL3 expression in 9-month-old TLR4M Tg mice may contribute to the increase in the cerebral Aβ load. It would be interesting to determine the role of CCL3 in cerebral β-amyloidosis by under- and over-expression of CCL3 in the brains of AD mouse models.

TLR2 deficiency (TLR2-/-) in an AD mouse model also increased soluble Aβ42 in the brain and exacerbated cognitive impairments [34]. Furthermore, injection of CpG oligodeoxynucleotides, a TLR9 ligand, reduced Aβ load in the brain and restored cognitive deficits in an AD mouse model [35,36]. These results suggest that activation of TLRs can be a therapeutic option for AD.

## Conclusion

In summary, our results suggest that TLR4 signaling is not involved in initiation of Aβ deposition and that microglia are activated and recruited in response to Aβ deposition via TLR4 signaling to promote Aβ clearance, resulting in protection of neurons from Aβ-mediated neurotoxicity. Because Aβ fibrils upregulate expression of CCL3 in myeloid cells through TLR4 activation, CCL3 may be involved in microglial recruitment and Aβ clearance. Thus, activation of microglia via TLR4 in early stages of AD pathogenesis is neuroprotective and TLR4 signaling pathways offer potential therapeutic targets.

## Abbreviations

AD: Alzheimer's disease; ANOVA: analysis of variance; APP: Aβ-protein precursor; BSA: bovine serum albumin; ELISA: enzyme-linked immunosorbent assay; GAPDH: Glyceraldehyde 3-phosphate dehydrogenase; IFN: interferon; IL: interleukin; PS1 and PS2: presenilin 1 and 2; PCR: polymerase chain reaction; SEM: standard error of the mean; TBS: tris-buffered saline; TGF: transforming growth factor; TNF: tumor necrosis factor; TLR: toll-like receptor.

## Competing interests

The authors declare that they have no competing interests.

## Authors' contributions

KF and RL designed the study and reviewed the data. MS, JJ, JL, JK, HK, RL and KF analyzed the data and wrote the manuscript. JJ, MS, JL, JK, AP, JAR, KT, and HK performed experiments. All authors have read and approved the final version of the manuscript.
